# Tea-induced improvement of endothelial function in humans: No role for epigallocatechin gallate (EGCG)

**DOI:** 10.1038/s41598-017-02384-x

**Published:** 2017-05-23

**Authors:** Mario Lorenz, Franziska Rauhut, Christine Hofer, Stefanie Gwosc, Eda Müller, Damaris Praeger, Benno F. Zimmermann, Klaus-Dieter Wernecke, Gert Baumann, Karl Stangl, Verena Stangl

**Affiliations:** 1 Medizinische Klinik für Kardiologie und Angiologie, Campus Mitte, Charité – Universitätsmedizin Berlin, 10117 Berlin, Germany; 2 Institute of Nutritional and Food Sciences, University of Bonn, 53117 Bonn, Germany; 3 Institut Kurz GmbH, 50829 Köln, Germany; 4 Charité – Universitätsmedizin Berlin und SOSTANA GmbH, 10318 Berlin, Germany; 5 DZHK (German Centre for Cardiovascular Research), partner site Berlin, 13347 Berlin, Germany

## Abstract

Consumption of tea is inversely associated with cardiovascular diseases. However, the active compound(s) responsible for the protective effects of tea are unknown. Although many favorable cardiovascular effects *in vitro* are mediated by epigallocatechin gallate (EGCG), its contribution to the beneficial effects of tea *in vivo* remains unresolved. In a randomised crossover study, a single dose of 200 mg EGCG was applied in three different formulas (as green tea beverage, green tea extract (GTE), and isolated EGCG) to 50 healthy men. Flow-mediated dilation (FMD) and endothelial-independent nitro-mediated dilation (NMD) was measured before and two hours after ingestion. Plasma levels of tea compounds were determined after each intervention and correlated with FMD. FMD significantly improved after consumption of green tea containing 200 mg EGCG (p < 0.01). However, GTE and EGCG had no significant effect on FMD. NMD did not significantly differ between interventions. EGCG plasma levels were highest after administration of EGCG and lowest after consumption of green tea. Plasma levels of caffeine increased after green tea consumption. The results show that EGCG is most likely not involved in improvement of flow-mediated dilation by green tea. Instead, other tea compounds, metabolites or combinations thereof may play a role.

## Introduction

Consumption of tea is associated with reduced progression of atherosclerosis and lower cardiovascular mortality^1, 2^. However, the substance(s) mediating the favorable cardiovascular effects of tea *in vivo* have not yet been identified. Tea contains high amounts of polyphenols with significant biological activities. The green tea catechin epigallocatechin gallate (EGCG) is the physiologically most active compound *in vitro* ^3^. A large number of *in vitro* studies and animal experiments provide evidence of the beneficial effects of EGCG on cardiovascular parameters^4, 5^. Stimulation of nitric oxide (NO) production by endothelial cells and subsequent NO-dependent vasodilation in isolated vessels are two of the most prominent cardiovascular actions of EGCG *in vitro* ^6–8^. Owing to promising results at the cellular level, EGCG has become extensively applied in human intervention studies. More than 80 studies with EGCG, which encompass a wide range of clinical applications, were registered on clinicaltrials.gov as of October 2016. It is largely unknown, however, whether the beneficial cardiovascular effects of EGCG *in vitro* can be reproduced in humans.

To elucidate this matter, we investigated the contribution of EGCG to tea-induced changes in flow-mediated dilation (FMD). Impaired endothelial function represents an early marker for later cardiovascular events^9^. It had been previously shown that consumption of green tea^10^ as well as of black tea^11^ improved FMD in humans. A recent meta-analysis demonstrated that short- and long-term tea consumption increases endothelial-dependent vasodilation^12^. The role of single tea compounds, and especially of EGCG, to the described cardiovascular effects, however, remains unclear. We studied the impact of a single dose of EGCG in three different application forms (green tea beverage, GTE, and isolated EGCG) on FMD in healthy volunteers. The amount of ingested EGCG (200 mg) corresponds to approx. 0.5 l of green tea. The results provide insights into the potential role of EGCG and other tea compounds in the beneficial cardiovascular effects of green tea *in vivo*.

## Results

### Interventions had no influence on plasma levels of cardiovascular risk markers

**Figure 1:**
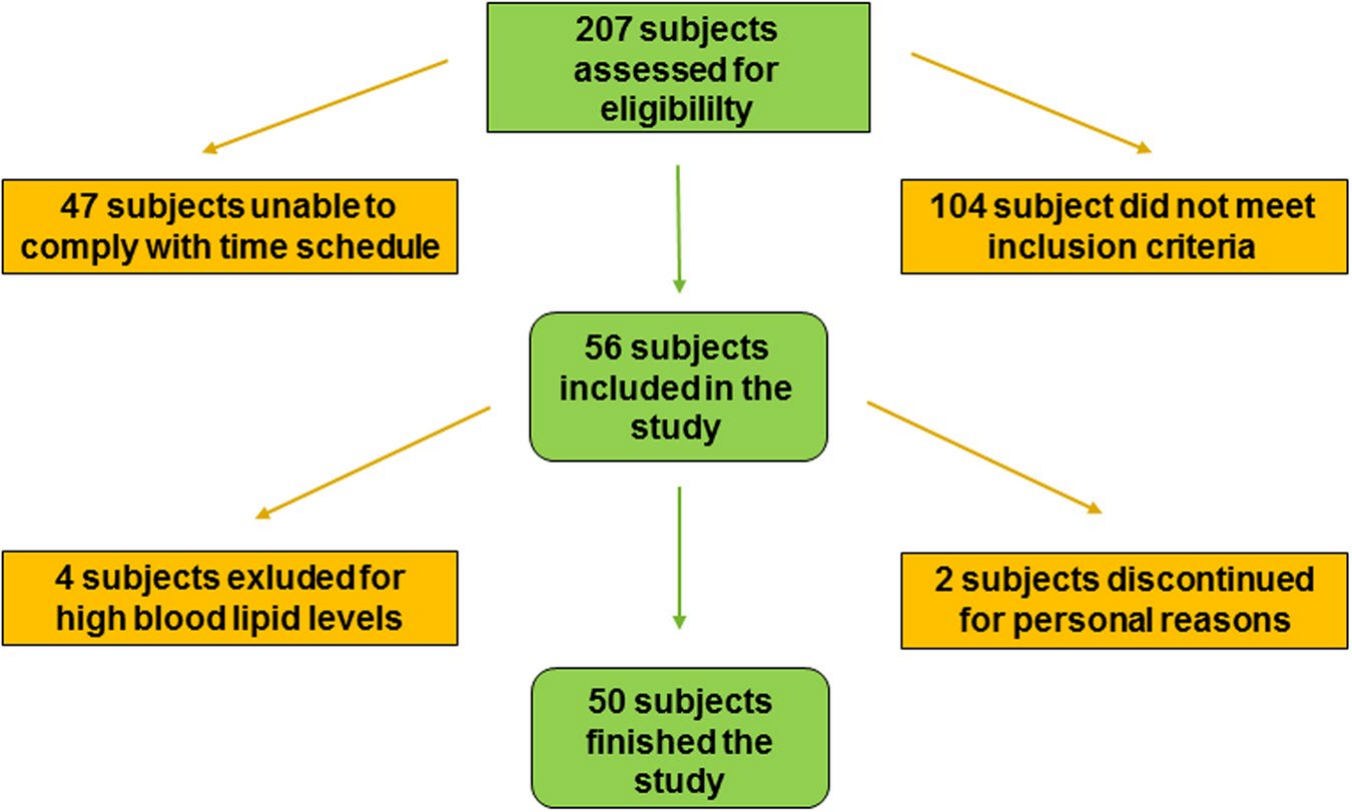
Flow-chart of the study.

A total of 207 men responded, and interviews were conducted with these volunteers to assess for eligibility. 56 subjects fulfilled the inclusion criteria, including the time requirements, and were enrolled in the study. Four participants were excluded during the study for having blood lipid levels outside the normal range. Two subjects dropped out for personal reasons. 50 subjects finished the study. A flow chart of the study is shown in Fig. 1. Table 1 summarizes the baseline characteristics of the study population. Subjects consumed a single dose of 200 mg EGCG as green tea beverage, as GTE, or as isolated EGCG. FMD was measured before and two hours after ingestion. To prevent food intake from interfering with EGCG absorption and FMD, the interventions were done in a fasting state. The composition of the interventions is shown in Table 2. Levels of LDL, HDL, total cholesterol, TG and CrP did not significantly change two hours after the interventions (Wilcoxon test, data not shown).

**Table 1:** Baseline characteristics of the study population (n = 50).

		Mean	SD
Age	(years)	33.9	7.6
SBP	(mm Hg)	122.0	9.4
DBP	(mm Hg)	69.7	7.6
BMI	(kg/m^2^)	23.7	2.5
HbA1_c_	(%)	5.2	0.3
Homocysteine	(µM)	11.3	4.7
CrP	(mg/l)	1.2	2.1
Total cholesterol	(mg/dl)	178.7	34.2
HDL cholesterol	(mg/dl)	54.3	11.4
LDL cholesterol	(mg/dl)	112.2	34.2
TG	(mg/dl)	93.2	51.1
Lipoprotein(a)	(mg/l)	153.2	277.7

SD, standard deviation; SBP, systolic blood pressure; DBP, diastolic blood pressure; BMI, body mass index; CrP, C-reactive protein; TG, triglycerides.

**Table 2:** Composition of the interventions.

	TB	GA	caffeine	GC	Catechin	GCG	EC	ECG	EGC	EGCG
Green tea (mg/l)	15.0	35.4	265	113	10.8	7.8	51.6	89.1	141	452.5
GTE (mg/g)	<0.01	13.2	7.1	<0.01	6.5	16.5	69.5	70.5	145	492.3
EGCG (mg/g)	<0.01	0.5	0.04	<0.01	0.7	<0.01	5.0	40.6	<0.01	936.0

TB, theobromine; GA, gallic acid; GC, gallocatechin; GCG, gallocatechin gallate; EC, epicatechin; ECG, epicatechin gallate; EGC, epigallocatechin; EGCG, epigallocatechin gallate; GTE, green tea extract.

### Only green tea improved FMD

**Figure 2:**
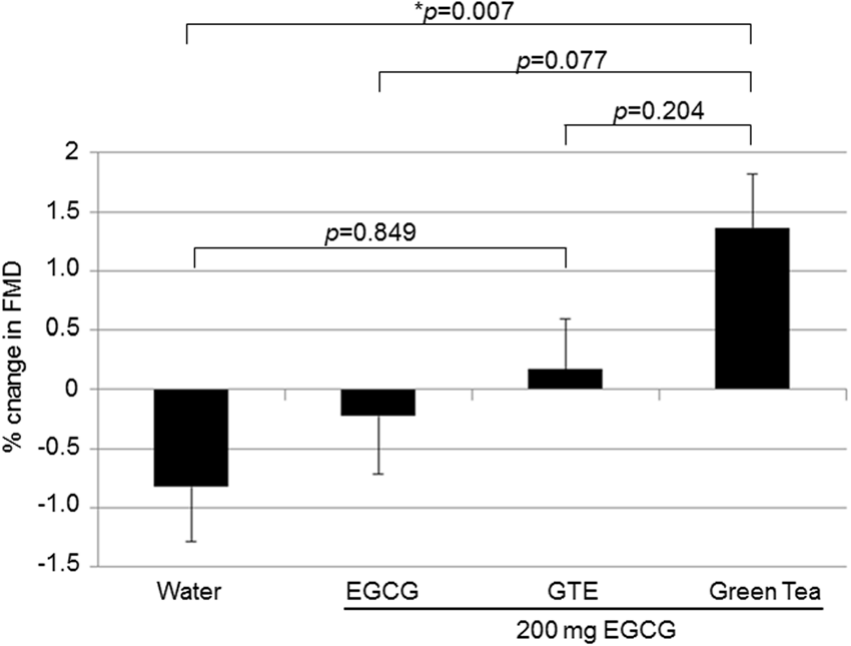
Only green tea increased flow-mediated dilation (FMD). Subjects consumed 200 mg of EGCG as isolated EGCG, GTE, or green tea after fasting overnight. An equal volume of hot water served as control. Green tea significantly increased FMD compared to GTE, EGCG, and water as control. Water slightly decreased FMD, whereas EGCG and GTE had little effects. Data are means ± SEM from n = 50 subjects. All p-values by repeated measures ANOVA followed by post hoc Bonferroni.

Before interventions, there were no statistical differences in baseline vessel diameter, in FMD, and in NMD between the four study arms by repeated measures ANOVA (Table 3). Hot water decreased FMD non-significantly by −0.84% (5.05 ± 2.65% at baseline and 4.21 ± 2.65% after two hours, p = 0.076, paired t-test). Green tea significantly improved FMD by 1.36% (4.49 ± 2.77% at baseline and 5.85 ± 3.61% after two hours, p = 0.004, paired t-test). GTE and EGCG, although containing the same amount of EGCG as the tea beverage, had no significant effect on FMD (GTE: 4.72 ± 3.16% at baseline and 4.90 ± 3.04% after two hours, p = 0.679, paired t-test; EGCG: 5.00 ± 3.07% at baseline and 4.77 ± 3.99% after two hours, p = 0.648, paired t-test). Between treatments, there were significant overall differences in changes of FMD (p = 0.006, repeated measures ANOVA). After post hoc Bonferroni analysis, only green tea significantly improved FMD compared to water (Fig. 2). No significant changes in FMD were observed between the other treatments. The overall effects on FMD increased with increasing complexity of the interventions – i.e., with an increasing number of compounds contained in the interventions (Fig. 2). There were significant overall differences in absolute FMD by repeated measures ANOVA two hours after ingestion between treatments (p = 0.032). After Bonferroni post hoc analysis, the absolute FMD differed significantly only between green tea and water (5.85 ± 3.61% after tea and 4.21 ± 2.65% after water, p = 0.017), whereas no significant differences were observed between the other interventions. The net response in FMD between green tea and water (mean FMD after green tea minus mean FMD after hot water as control) was 1.64%.

**Table 3:** Baseline diameter and baseline FMD and NMD before interventions.

	Water	EGCG	GTE	Green Tea
Baseline diameter FMD (mm)	4.07 (0.49)	4.08 (0.51)	4.05 (0.48)	4.11 (0.51) n.s.
FMD (%)	5.05 (2.65)	5.00 (3.07)	4.72 (3.16)	4.49 (2.77) n.s.
Baseline diameter NMD (mm)	4.06 (0.50)	4.06 (0.53)	4.08 (0.48)	4.08 (0.50) n.s.
NMD (%)	19.57 (6.40)	18.19 (5.59)	18.82 (6.07)	19.70 (6.14) n.s.

FMD, flow-mediated dilation; NMD, nitro-mediated dilation; GTE, green tea extract; baseline diameter, diameter of artery brachialis before hyperaemic stimulus or application of nitroglycerine. n.s.; no significant differences by repeated measures ANOVA. Values are mean (SD) from n = 50 subjects.

### NMD was not affected by the interventions

Changes in endothelial-independent NMD did not significantly differ between interventions (water: −0.32 ± 5.42%, EGCG: −0.15 ± 4.78%, GTE: −0.36 ± 4.71%, green tea: −0.79 ± 5.00%; p = 0.92, repeated measures ANOVA), and did not coincide with FMD.

### EGCG plasma levels did not correlate with changes in FMD

**Figure 3:**
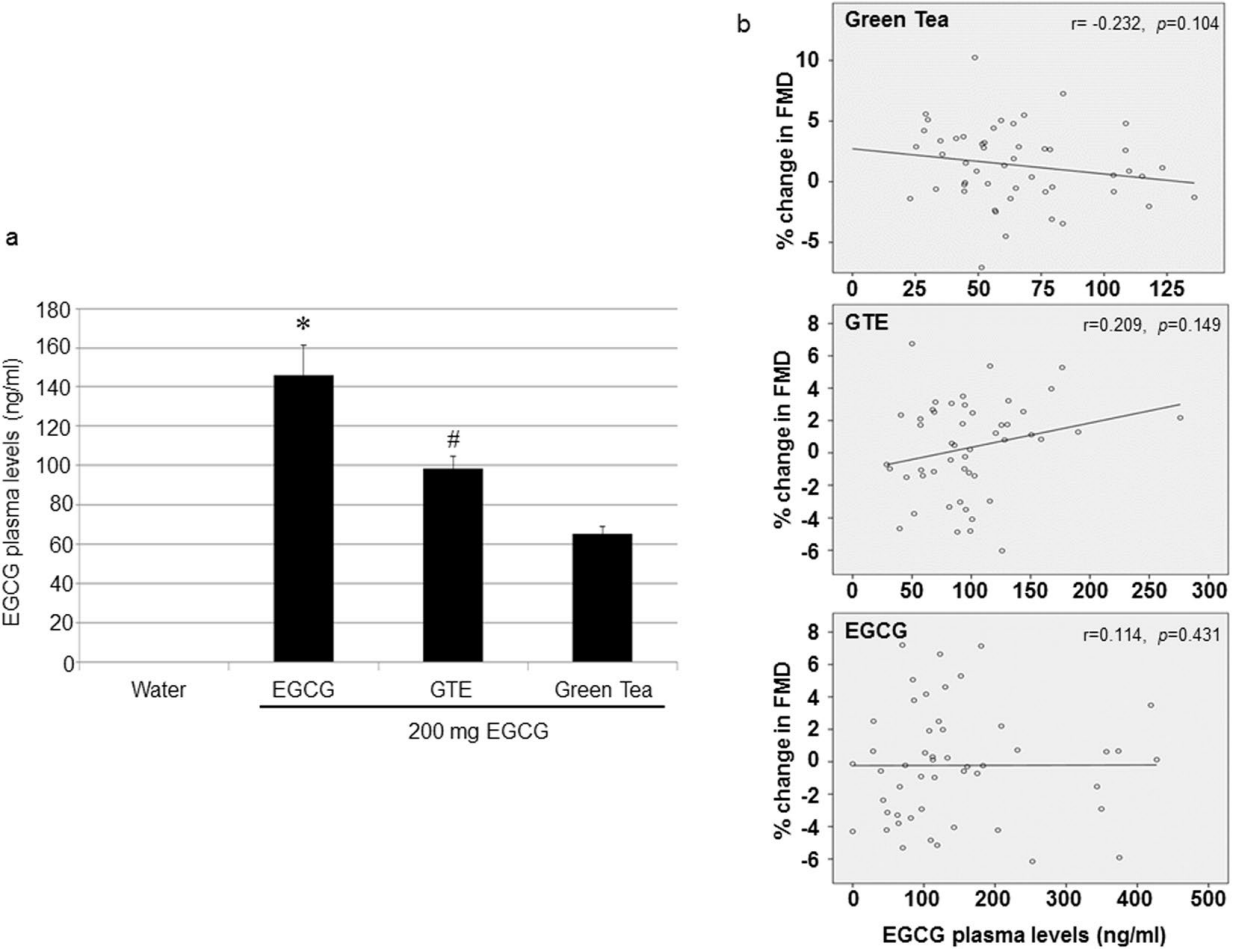
EGCG plasma levels did not correlate with changes in FMD. Shown are the sum of free and conjugated EGCG plasma levels two hours after consumption (**a**). Data are means ± SEM from n = 50 subjects for water, EGCG, and green tea and n = 49 for GTE. *p < 0.05 versus GTE and green tea, ^#^p < 0.05 versus EGCG and green tea (Wilcoxon test). Correlations of EGCG plasma levels with changes in FMD after green tea, GTE, and EGCG (**b**). There were no significant correlations between EGCG plasma levels and FMD for all treatments (Spearman’s). Data are from n = 50 subjects for EGCG and green tea and n = 49 for GTE.

Plasma levels of EGCG and of the other tea catechins were below detection limits before interventions and after consumption of hot water (data not shown). The highest EGCG plasma levels were observed after intake of isolated EGCG and the lowest after consumption of green tea. Administration of GTE resulted in intermediate EGCG plasma levels (Fig. 3a). EGCG was present in the free (unconjugated) form in human plasma, in agreement with previous studies^13^. No significant correlations between EGCG plasma levels and changes in FMD were observed for all interventions (Fig. 3b).

### No correlations of catechin plasma levels with FMD

**Figure 4:**
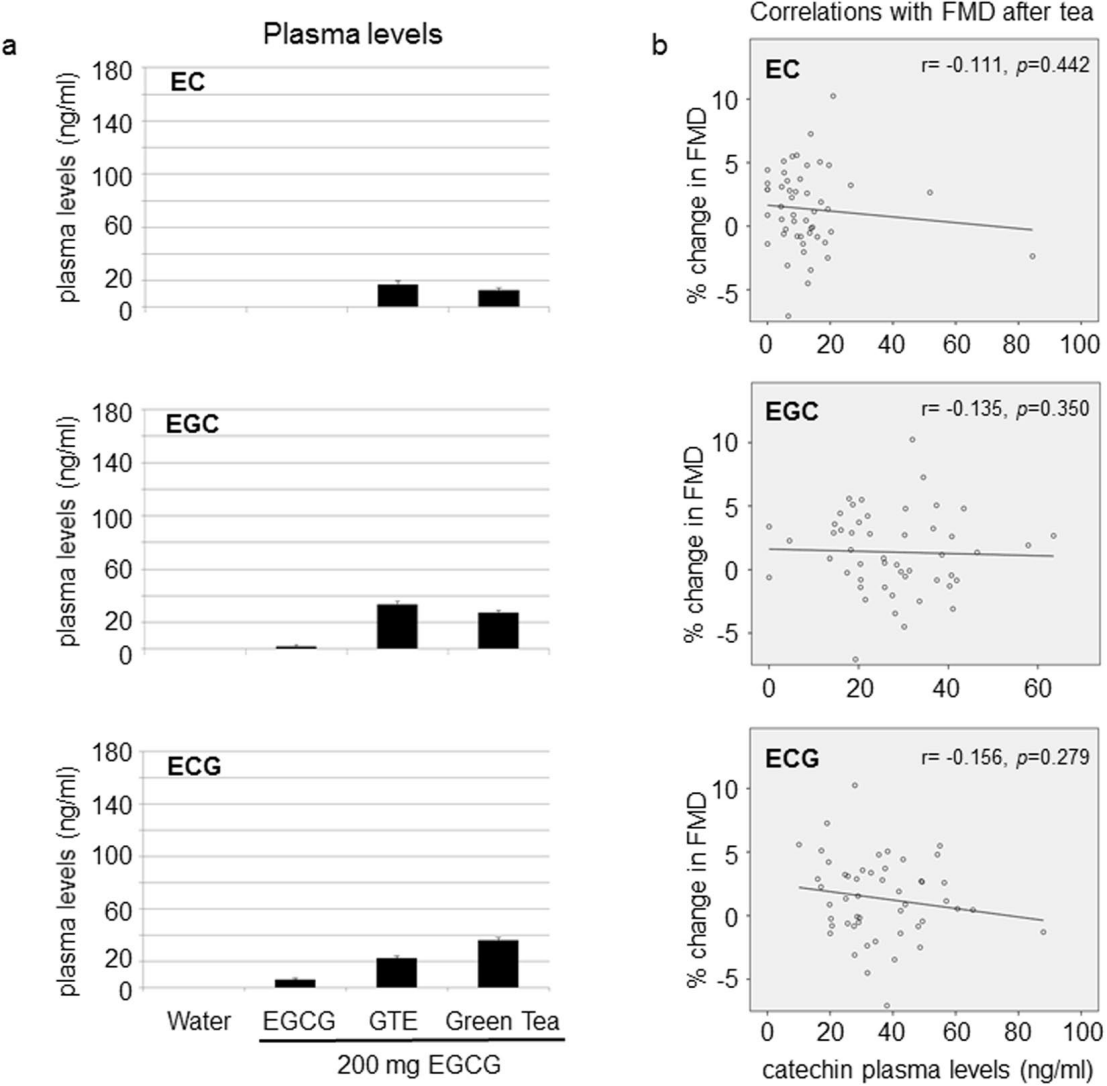
No correlations of catechin plasma levels with changes in FMD. Plasma levels of EC (epicatechin), EGC (epigallocatechin), and ECG (epicatechin gallate) after hydrolysis (sum of free and conjugated catechins) two hours after consumption (**a**). The scale of the *y* axes are equal to Fig. 3 to facilitate comparisons with EGCG levels. Data are means ± SEM from n = 50 subjects for water, EGCG, and green tea and n = 49 for GTE. Correlations of catechin plasma levels with FMD changes after tea consumption (**b**). Non-significant negative correlations were observed between catechin plasma levels and FMD changes (Spearman’s). Data are from n = 50 subjects.

Beneficial effects on endothelial function have also been described for epicatechin (EC)^14^. EC plasma levels were higher after GTE and lower after green tea (Fig. 4a), and no correlation with changes in FMD was observed after green tea consumption (Fig. 4b). Similar results were obtained for epigallocatechin (EGC) (Fig. 4a,b). Mean plasma concentrations for epicatechin gallate (ECG) were very low, and a negative correlation with changes in FMD after green tea was observed (Fig. 4a,b). The sum of all catechins (total catechin plasma levels) showed a non-significant negative correlation with changes in FMD after tea (r = −0.239, p = 0.095, Spearman’s). An overview of catechin plasma levels after the interventions before and after deconjugation is given in Table 4.

**Table 4:** Catechin plasma levels before and after hydrolysis (deconjugation) 2 hours after interventions.

	EC				ECG				EGC				EGCG			
	before hydrolysis		after hydrolysis		before hydrolysis		after hydrolysis		before hydrolysis		after hydrolysis		before hydrolysis		after hydrolysis	
	ng/ml	µM	ng/ml	µM	ng/ml	µM	ng/ml	µM	ng/ml	µM	ng/ml	µM	ng/ml	µM	ng/ml	µM
Green tea	n.d.	n.d.	12.6 (13.5)	0.03 (0.03)	34.1 (15.0)	0.07 (0.03)	35.8 (15.1)	0.08 (0.03)	1.7 (4.3)	n.q.	27.3 (12.8)	0.06 (0.03)	66.9 (27.0)	0.15 (0.06)	65.2 (28.0)	0.14 (0.06)
GTE	n.d.	n.d.	17.2 (15.6)	0.04 (0.03)	21.7 (10.9)	0.05 (0.02)	22.3 (10.7)	0.05 (0.02)	2.4 (4.9)	0.01 (0.01)	33.6 (14.8)	0.07 (0.03)	102.7 (47.2)	0.22 (0.10)	98.2 (45.6)	0.21 (0.10)
EGCG	n.d.	n.d.	n.d.	n.d.	5.9 (8.9)	0.01 (0.02)	5.8 (8.8)	0.01 (0.02)	0.5 (3.3)	n.q.	1.8 (6.7)	n.q.	149.0 (112.0)	0.32 (0.24)	145.9 (109.6)	0.32 (0.24)

EC, epicatechin; ECG, epicatechin gallate; EGC, epigallocatechin; EGCG, epigallocatechin gallate; n.d. not detectable; n.q. not quantifiable.

Data are mean (SD) from n = 50 subjects for green tea and EGCG and n = 49 for GTE.

### Caffeine plasma levels increased after green tea consumption

**Figure 5:**
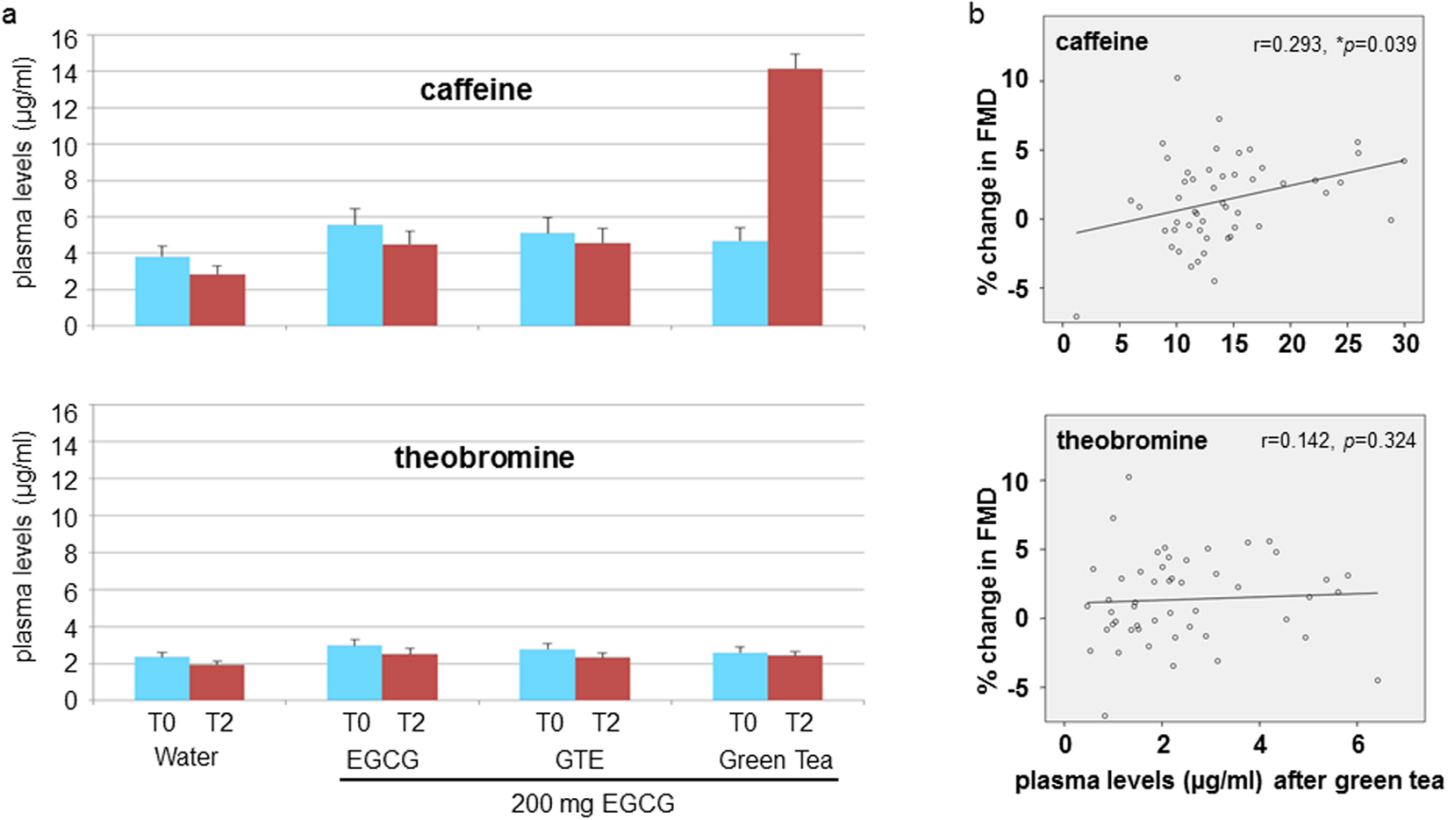
Caffeine but not theobromine plasma levels correlate with FMD changes after green tea consumption. Plasma levels of caffeine and theobromine before and two hours after interventions (**a**). For comparison, the scale is identical for both compounds. Data are means ± SEM from n = 50 subjects for water, EGCG, and green tea and n = 49 for GTE. Significant positive correlations between plasma levels and changes in FMD after green tea consumption were obtained for caffeine, but not for theobromine (Spearman’s) (**b**). Data are from n = 50 subjects.

Since catechin plasma levels were not sufficient to account for differential effects on FMD between interventions, we measured blood levels of additional tea compounds. Plasma levels of caffeine markedly increased two hours after consumption of green tea, whereas a slight decrease was observed in the other interventions (Fig. 5a). Plasma levels of theobromine slightly decreased after all interventions (Fig. 5a). Caffeine and theobromine were also present at baseline (before intervention) in all treatments. We observed a slight, but significant positive correlation between plasma concentrations of caffeine and changes in FMD after green tea consumption, whereas plasma levels of theobromine did not correlate with FMD (Fig. 5b).

## Discussion

This study shows for the first time that EGCG is most likely not involved in tea-induced improvement of endothelial function in humans. Although EGCG plasma levels were highest after intake of EGCG and lowest after consumption of green tea, only green tea improved FMD. It has been consistently demonstrated that green tea improves endothelial function in humans^10, 15, 16^. The results of our study confirmed these findings. Although not statistically significant, a minor effect on FMD was also observed for GTE in our study.

**Figure 6:**
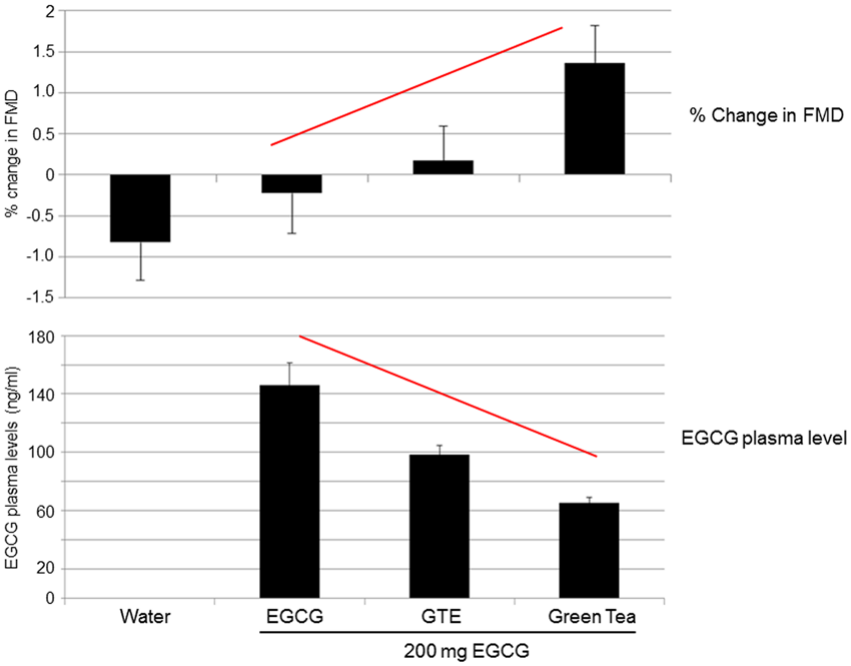
Inverse relationship between FMD and EGCG plasma levels after the interventions. FMD gradually increased with the number of compounds present (i.e., with the complexity) of the intervention (upper panel), but EGCG plasma levels are inversely proportional.

Green tea and EGCG stimulated NO production in endothelial cells by activating endothelial nitric oxide synthase (eNOS) and induced vasodilation in aortic rings^10, 17^. However, the contribution of EGCG to the effects of green tea *in vivo* remains elusive. In our study, we observed no effect of 200 mg isolated EGCG on endothelial function in humans. Widlansky *et al*. found an acute (but not long-term) improvement in FMD with 300 mg EGCG in patients with coronary artery disease^18^. However, apart from different EGCG doses used, in contrast to our study Widlansky *et al*. observed no differences in the absolute FMD between EGCG and placebo after supplementation. A numerical lower baseline FMD before EGCG as compared to before placebo could have contributed to their results. *In vitro*, significant effects of EGCG on NO production and vasodilation were obtained only at concentrations of 1 µM or higher^6–8^, concentrations that were not achieved in plasma in our study and in Widlansky *et al*.^18^. Surprisingly, mean plasma levels of EGCG in our study were inversely associated with changes in FMD between interventions (Fig. 6). All of the above results make a substantial contribution of EGCG to the vasodilating effects of green tea *in vivo* very unlikely.

Stimulation of endothelial NO production and vasodilation in isolated vessels have also been described for EC^8, 19^. In a small study by Schroeter *et al*., the administration of 1–2 mg/kg body weight of EC resulted in improved FMD in three subjects^14^. However, EC supplementation for 4 weeks (100 mg/d) had no significant effects on FMD in 35 subjects^20^. Consumption of 442 ml green tea in our study resulted in the intake of 23 mg EC, which is much less than the amount of EC ingested in the study by Schroeter *et al*. EC plasma levels in our study were very low and did not parallel changes in FMD between interventions. In line with previous reports^13^, EC was fully conjugated and not present in the free form in plasma. Therefore, the involvement of EC in the effects of green tea on FMD is rather unlikely. Mean plasma levels of the other catechins ECG and EGC were also very low and showed no correlations with FMD, indicating that both catechins are in all likelihood not involved in the improvement of FMD by green tea. Also, the sum of all catechins showed no correlation with FMD.

What other compounds could potentially contribute to the effects of green tea? Caffeine is present in high amounts in green and black tea. Consequently, plasma levels of caffeine substantially increased after consumption of green tea in our study. However, the published data on the role of caffeine on flow-mediated dilation are conflicting. A cup of decaffeinated coffee had no effect on FMD in healthy adults, whereas caffeinated coffee (with 80 mg of caffeine) even declined FMD^21^. In a similar study, decaffeinated coffee resulted in a slight increase in FMD, whereas caffeinated coffee significantly decreased FMD in healthy subjects^22^. Consumption of green tea significantly improved FMD, however, the corresponding amount of caffeine contained in this tea (125 mg) did only slightly increase FMD^16^. Likewise, a significant improvement of FMD was observed two hours after consumption of black tea, but an equivalent dose of 200 mg caffeine had no effect^11^. But there is also evidence that caffeine exerts beneficial effects on endothelial function. A single dose of caffeine acutely improved FMD in patients with stable CAD, and increased forearm blood flow^23, 24^. Chronic coffee consumption was associated with higher FMD^25^, and moderate coffee consumption resulted in a decreased risk of cardiovascular diseases^26^. An improved FMD was also observed after administration of a green tea extract containing caffeine^27^. In addition, an inverse association with risk of CVD and stroke was shown for higher coffee and green tea consumption^28^. Tea (mainly black) as well as moderate coffee consumption have been shown to decrease the risk for coronary heart disease^2^. Taken together, these studies point to a cardiovascular risk reduction for both tea and coffee.

In black tea, the amount of EGCG (and of other catechins) is much lower compared to green tea, depending on the degree of fermentation. Theaflavins and thearubigins, the major polyphenolic compounds in black tea, are too large for substantial absorption into the circulation^29^. Green and black tea are, however, equally effective in the improvement of FMD^10, 12^, and both teas contain a similar amount of caffeine. However, other compounds including plasma metabolites of tea polyphenols, such as methylated compounds/phase I-breakdown or gut microbial metabolites (in addition to glucuronidated and sulphated substances), could also be involved. Although EGCG can be methylated^30^, very low^31^, or no methylated (or otherwise metabolised) EGCG^32^ was found in human plasma after consumption of a green tea extract or green tea. An inverse association between the degree of urinary flavonoid O-methylation (4OMGA) and FMD after consumption of black tea indicates that methylation of tea flavonoids could rather affect endothelial function^33^. However, the influence of other (as yet unknown) phase I-breakdown or gut microbial metabolites on tea-induced FMD cannot be fully excluded.

Some study limitations should be acknowledged. The observed correlation between caffeine plasma levels and FMD is only weak and does not prove a role of caffeine in tea-induced endothelial function. A study with caffeine and hot water, EGCG and green tea or with caffeinated and decaffeinated green tea would be required to verify the role of caffeine in tea-induced FMD. Although EGCG did not mediate the effects of green tea on FMD in our study with healthy subjects, a contribution of EGCG to tea-mediated improvements of endothelial dysfunction cannot be fully excluded. Even though the amount of EGCG used in our study (200 mg) corresponds to a nutritionally relevant dose that can be consumed by drinking tea, it does not necessarily reflect habitual tea drinking of several cups per day. Also, it should be noted that the contributions of single tea compounds to tea-induced FMD cannot be generalised for other cardiovascular, neurological, or anti-tumor effects of green tea.

In summary, our data indicate that EGCG is not involved in changes of FMD by green tea. The lack of an impact of EGCG on tea-induced improvements in endothelial function clearly points out that caution is indicated when translating promising *in vitro* experiments using a single isolated compound from a food matrix into the human situation. In light of many ongoing human studies with EGCG it will be of considerable interest to study the contribution of this promising *in vitro* candidate to other putative beneficial health effects of green tea.

## Methods

### Study population

Healthy men between 20 and 50 years were recruited by press advertisements. Subjects with chronic diseases and known cardiovascular risk factors such as high blood cholesterol (≥240 mg/dl), diabetes, arterial hypertension, and body mass index >27 kg/m^2^ were excluded. Plasma lipid profiles and blood pressure were required to be within the normal range for study inclusion. Regular tea consumption, current smoking, chronic drug use, and alcohol abuse were additional exclusion criteria. The study was approved by the Charité University Hospital Ethics Committee and participants provided their written informed consent. The study protocol was in accordance to local university guidelines and with the principles outlined in the Declaration of Helsinki.

### Study design

This pilot study was a prospective, exploratory, randomised, crossover study. A single dose of 200 mg of EGCG was orally ingested in different application forms. After fasting overnight, each subject consumed 200 mg of EGCG on different days, either as green tea beverage, green tea extract (GTE), or purified EGCG. The same amount of hot water was applied with each of the EGCG forms ingested. The corresponding amount of hot water served as control. Randomization was performed using computer-generated random numbers for the order of treatments. The interventions were at least 3 days apart. Before and two hours after ingestion, flow-mediated and nitro-mediated dilation of the brachial artery was assessed by vascular ultrasound. Blood was taken before and two hours after intervention, and plasma levels of tea compounds were measured. The study was performed at the Department of Cardiology and Angiology (Campus Mitte) at Charité – University Medicine Berlin, Germany. The study has been registered under ClinicalTrials.gov https://clinicaltrials.gov/ct2/show/NCT01662232, registration No. NCT01662232; date of registration: August 7, 2012.

### Interventions

Darjeeling green tea was obtained from King’s Teagarden, Berlin, Germany. For determination of the EGCG content of the beverage before the start of the study, 6.75 grams of ground tea solids were brewed with 550 ml of boiling water for 3 min. The infusion had an EGCG content of 452.5 ± 8.7 mg/l (mean ± SD, n = 6). For administration of 200 mg of EGCG, the participants consumed 442 ml of the beverage. The GTE was supplied by Nutri-Fit GmbH & Co. KG. The EGCG content of the extract was 492.3 ± 1.4 mg/g (n = 3). For ingestion of 200 mg of EGCG, the subjects took a capsule containing 406.3 mg of GTE. The purity of the EGCG (kindly provided by Nutri-Fit GmbH & Co. KG) as determined by HPLC prior to the study was 93.6 ± 1.3% (n = 3). To correct for EGCG content, the subjects took a capsule containing 214 mg of EGCG for ingestion of 200 mg EGCG. An equal amount of hot water (442 ml, according to the volume of the beverage) was applied together with all capsules. 442 ml of hot water served as control.

### Assessment of flow-mediated (FMD) and nitro-mediated dilation (NMD)

Endothelium-dependent FMD was assessed by measuring the change in brachial artery diameter during reactive hyperemia after cuff occlusion of the forearm, according to established guidelines^34^. Endothelial function was measured by high-resolution vascular ultrasound using Vivid 7 (General Electric Medical Systems) with a 12-MHz linear array transducer. We performed all measurements in the morning at the same time of the day. Data were analysed using the automatic edge detection software FMD Studio Cardiovascular Suite (CVS 2.0, QUIPU, Pisa, Italy), which analyses the data in real time^35^. Baseline diameter was assessed for 1 min, followed by 5 min of cuff occlusion and determination of diameter changes after reactive hyperemia for 4 min after cuff release. FMD was defined as the maximum percentage change in diameter compared with baseline measurements. Percent changes in FMD of the brachial artery were determined before and two hours after each intervention. Endothelium-independent vasodilation (NMD) was measured 10 min after FMD by sublingual application of nitroglycerin spray (0.4 mg) before and after each intervention. Baseline diameter was assessed for 1 min, and the increase in diameter of the brachial artery after application of nitroglycerin was measured for 9 min.

### Blood parameters

For cardiovascular risk assessment, the following parameters were measured by conventional laboratory methods: total cholesterol, LDL cholesterol, HDL cholesterol, triglycerides (TG), C-reactive protein (CrP), lipoprotein(a), HbA1c, and homocysteine. Total cholesterol, LDL cholesterol, HDL cholesterol, triglycerides, and CrP were determined before and two hours after each intervention.

### Composition of the interventions and determination of plasma levels of tea compounds

Concentrations of the individual tea substances in the interventions were measured on a Waters Acquity UPLC (Waters, Milford, MA, USA). The equipment consists of a binary pump (BSM), an autosampler (SM) cooled at 10 °C, a column oven (CM) set at 40 °C, a diode array detector (PDA) scanning from 190 to 500 nm, and an Acquity TQD triple-quadrupole mass spectrometer with an electrospray interface. A Waters BEH phenyl column (50 mm x 2.1 mm, 1.7 µm) with a VanGuard precolumn was employed at a flow rate of 0.6 ml/min. The eluents acetonitrile/0.1% formic acid (A) and water/0.1% formic acid (B) were run with the following gradient: 0 min: 6% A; 1.5 min 13% A; 2.3–3.3 min: 100% A; 4.3–5.3 min: 6% A. Peak identity was confirmed by MS/MS. For quantification, authentic reference compounds were used (detection wavelength 278 nm) for external calibration. Liquid samples were adequately diluted, solid samples were dissolved with methanol/water (80/20), and filtered through 0.2 µm Chromafil RC-20/15 MS filters (Macherey-Nagel, Düren, Germany).

Plasma levels of green tea catechins were measured as previously described^13^. To determine the levels of free (unconjugated) catechins, all samples were analysed in parallel, with omission of enzymatic hydrolysis. Plasma levels of caffeine and theobromine were determined as described^36^, with slight modifications: 40 µl of 20% aqueous perchloric acid were added to 100 µl of thawed plasma samples and centrifuged. The supernatant was injected into the UHPLC system (as described above). For external calibration, authentic reference compounds were used (detection wavelength 278 nm).

### Statistical analysis

For this exploratory pilot study, comparisons of parameters between the four interventions water, EGCG, GTE, and green tea were performed by repeated measures ANOVA. After overall statistical differences between treatments (p < 0.05), this was followed by post hoc Bonferroni correction to adjust for multiple testing. Pairwise comparisons of parameters before and after interventions within treatments were performed using paired t-tests or Wilcoxon tests (for non-Gaussian distributions). Correlations between plasma levels of individual tea substances and FMD were calculated by using Spearman’s rho coefficient. All statistical tests were two-sided, with the level of significance accepted at p < 0.05. Statistical analysis was performed using SPSS, release 22.0 (SPSS, Inc., Chicago, IL, USA). Values are given as means±SD.

## References

[1] Kuriyama S (2006). Green tea consumption and mortality due to cardiovascular disease, cancer, and all causes in Japan: the Ohsaki study. JAMA.

[2] de Koning Gans JM (2010). Tea and coffee consumption and cardiovascular morbidity and mortality. Arterioscler. Thromb. Vasc. Biol..

[3] Lorenz M (2013). Cellular targets for the beneficial actions of tea polyphenols. Am. J. Clin. Nutr..

[4] Chyu KY (2004). Differential effects of green tea-derived catechin on developing versus established atherosclerosis in apolipoprotein E-null mice. Circulation.

[5] Stangl V, Dreger H, Stangl K, Lorenz M (2007). Molecular targets of tea polyphenols in the cardiovascular system. Cardiovasc. Res..

[6] Lorenz M (2004). A constituent of green tea, epigallocatechin-3-gallate, activates endothelial nitric oxide synthase by a phosphatidylinositol-3-OH-kinase-, cAMP-dependent protein kinase-, and Akt-dependent pathway and leads to endothelial-dependent vasorelaxation. J. Biol. Chem..

[7] Kim JA (2007). Epigallocatechin gallate, a green tea polyphenol, mediates NO-dependent vasodilation using signaling pathways in vascular endothelium requiring reactive oxygen species and Fyn. J. Biol. Chem..

[8] Aggio A (2013). Endothelium/nitric oxide mechanism mediates vasorelaxation and counteracts vasoconstriction induced by low concentration of flavanols. Eur. J. Nutr..

[9] Ras RT, Streppel MT, Draijer R, Zock PL (2013). Flow-mediated dilation and cardiovascular risk prediction: a systematic review with meta-analysis. Int. J. Cardiol..

[10] Jochmann N (2008). The efficacy of black tea in ameliorating endothelial function is equivalent to that of green tea. Br. J. Nutr..

[11] Duffy SJ (2001). Short- and long term black tea consumption reverses endothelial dysfunction in patients with coronary artery disease. Circulation.

[12] Ras RT, Zock PL, Draijer R (2011). Tea consumption enhances endothelial-dependent vasodilation; a Meta-analysis. PLoS ONE.

[13] Zimmermann BF (2009). A shortcut from plasma to chromatographic analysis: straight-forward and fast sample preparation for analysis of green tea catechins in human plasma. J. Chromatogr. B Analyt. Technol. Biomed. Life Sci..

[14] Schroeter H (2006). (−)-Epicatechin mediates beneficial effects of flavanol-rich cocoa on vascular function in humans. Proc. Natl. Acad. Sci. USA.

[15] Nagaya N (2004). Green tea reverses endothelial dysfunction in healthy smokers. Heart.

[16] Alexopoulos N (2008). The acute effect of green tea consumption on endothelial function in healthy individuals. Eur. J. Cardiovasc. Prev. Rehabil..

[17] Lorenz M (2009). Green and black tea are equally potent stimuli of NO production and vasodilation: new insights into tea ingredients involved. Basic Res. Cardiol..

[18] Widlansky ME (2007). Acute EGCG supplementation reverses endothelial dysfunction in patients with coronary artery disease. J. Am. Coll. Nutr..

[19] Ramirez-Sanchez I, Maya L, Ceballos G, Villarreal F (2010). (−)-epicatechin activation of endothelial cell endothelial nitric oxide synthase, nitric oxide, and related signaling pathways. Hypertension.

[20] Dower JI (2015). Effects of the pure flavonoids epicatechin and quercetin on vascular function and cardiometabolic health: a randomized, double-blind, placebo-controlled, crossover trial. Am. J. Clin. Nutr..

[21] Papamichael CM (2005). Effect of coffee on endothelial function in healthy subjects: the role of caffeine. Clin. Sci. (Lond)..

[22] Buscemi S (2010). Acute effects of coffee on endothelial function in healthy subjects. Eur. J. Clin. Nutr..

[23] Shechter M (2011). Impact of acute caffeine ingestion on endothelial function in subjects with and without coronary artery disease. Am. J. Cardiol..

[24] Umemura T (2006). Effects of acute administration of caffeine on vascular function. Am. J. Cardiol..

[25] Siasos G (2013). Consumption of a boiled Greek type of coffee is associated with improved endothelial function: the Ikaria study. Vasc. Med..

[26] Ding M, Bhupathiraju SN, Satija A, van Dam RM, Hu FB (2014). Long-term coffee consumption and risk of cardiovascular disease: a systematic review and a dose-response meta-analysis of prospective cohort studies. Circulation.

[27] Tinahones FJ (2008). Green tea reduces LDL oxidability and improves vascular function. J. Am. Coll. Nutr..

[28] Kokubo Y (2013). The impact of green tea and coffee consumption on the reduced risk of stroke incidence in Japanese population: the Japan public health center-based study cohort. Stroke.

[29] Clifford MN, van der Hooft JJ, Crozier A (2013). Human studies on the absorption, distribution, metabolism, and excretion of tea polyphenols. Am. J. Clin. Nutr..

[30] Lu H, Meng X, Yang CS (2003). Enzymology of methylation of tea catechins and inhibition of catechol-O-methyltransferase by (−)-epigallocatechin gallate. Drug Metab. Dispos.

[31] Miller RJ (2012). A preliminary investigation of the impact of catechol-O-methyltransferase genotype on the absorption and metabolism of green tea catechins. Eur. J. Nutr..

[32] Stalmach A, Troufflard S, Serafini M, Crozier A (2009). Absorption, metabolism and excretion of Choladi green tea flavan-3-ols by humans. Mol. Nutr. Food Res..

[33] Hodgson JM, Puddey IB, Burke V, Croft KD (2006). Is reversal of endothelial dysfunction by tea related to flavonoid metabolism?. Br. J. Nutr..

[34] Thijssen DH (2011). Assessment of flow-mediated dilation in humans: a methodological and physiological guideline. Am. J. Physiol. Heart Circ. Physiol..

[35] Faita F (2011). Comparison of two automatic methods for the assessment of brachial artery flow-mediated dilation. J. Hypertens..

[36] Schreiber-Deturmeny E, Bruguerolle B (1996). Simultaneous high-performance liquid chromatographic determination of caffeine and theophylline for routine drug monitoring in human plasma. J. Chromatogr. B Biomed. Appl..

